# The therapeutic value of cerebrospinal fluid ctDNA detection by next-generation sequencing for meningeal carcinomatosis: a case report

**DOI:** 10.1186/s12883-019-1266-x

**Published:** 2019-03-09

**Authors:** Xiaosu Guo, Junzhao Cui, Yue Zhao, Weixin Han, Yueli Zou, Ruiping Gao, Qing Li, Xiaoqing Li, Junying He, Hui Bu

**Affiliations:** Department of Neurology, The Second Hospital of Hebei Medical University, No 215, Peace Road, Shijiazhuang, China

**Keywords:** Meningeal carcinomatosis, Cerebrospinal fluid, ctDNA, Next generation sequencing, Drug treatment

## Abstract

**Background:**

It is usually very complicated to treat meningeal carcinomatosis, and it is important to treat it as soon as possible.

**Case presentation:**

The 19-Del mutation was found in the exon for the epidermal growth factor receptor gene in the pleural effusion of a patient on March 11th, 2015. He took 250 mg of oral gefitinib once a day for 11 months beginning in December of 2015. On the 3rd of November 2016, he arrived at the hospital and presented with dizziness, headache and transient blurred vision. At this time, he began to take 4 mg of oral zoledronic acid once a month to prevent bone metastases. The result of a cytology exam of the cerebrospinal fluid showed that the man had meningeal carcinomatosis. The 19-Del mutation and the 20-T790 M mutation in the exon of the epidermal growth factor receptor gene was found by the next generation sequencing of the CSF. Then, he discontinued taking gefitinib and began to take 90–100 mg of oral AZD9291 once a day in November 2016. After adjusting the medication dose based on the NGS, his headache was noticeably reduced, and his condition gradually stabilized.

**Conclusions:**

Cerebrospinal fluid ctDNA detection by next generation sequencing may become a suitable biomarker to monitor clinical treatment response in meningeal carcinomatosis.

## Background

Meningeal carcinomatosis (MC) is a rare complication of extraneural solid tumors and consists of a focal or multiple malignant infiltration of neoplastic cells in the leptomeninges of the brain or spinal cord [1]. It is well recognized that MC occurs in 1–5% of patients with solid tumors. It is reported that 5% of patients with breast cancer, 23% of patients with melanoma and 9–25% of patients with small-cell lung cancer can develop MC [1]. Some clinical symptoms of MC include headache and nonspecific nerve palsy, and the patient’s condition can deteriorate quite quickly. In addition, MC is usually fatal to patients with advanced cancer with a mean survival time of 4 to 6 weeks. Early treatment is usually very complicated. Although EANO-ESMO clinical practice guidelines of leptomeninges of metastasis have been presented [2], the recommendations based on the evidence are mostly based on expert opinion with a low level of consensus. Given the limitations in the diagnosis and treatment of MC, missed diagnoses and delayed treatment are inevitable. Therefore, it is urgent to find a potential and effective treatment for MC.

Circulating tumor DNA (ctDNA) consists of short, double stranded DNA fragments that are released by tumors. It has been shown that plasma ctDNA can be used to characterize and monitor tumors [3–6]. ctDNA is a quantitative measure, and the concentration at a given time-point appears to be generally proportional to tumor progression [6–8]. Generally, ctDNA can be measured from different noninvasive sources, such as blood plasma, sputum, urine, or cerebrospinal fluid (CSF). Therefore, this enables convenient testing and real time monitoring of disease dynamics. There are various applications of ctDNA analysis, including therapy response monitoring, identification of resistance mechanisms, and relapse prediction. It has been noted, that next-generation sequencing (NGS) based ctDNA analysis has been able to identify epidermal growth factor receptor (*EGFR*) mutations in plasma with high specificity and sensitivity.

CSF, which can have intimate contact with tumor cells, is an easily obtained body fluid that reflects the underlying pathological state of the central nervous system. Recently, ctDNA has been found in the CSF of patients with brain tumors [9, 10]. Additionally, a case report underscored the potential of CSF-derived ctDNA analysis in the management of patients with human epidermal growth factor receptor-positive breast cancer [11]. It has been found that CSF ctDNA levels longitudinally fluctuate and follow changes in brain tumor burden variations, which provides a biomarker for monitoring brain malignancies [12]. Therefore, CSF ctDNA levels can follow tumor development, which is more sensitive and informative than traditional imaging. Moreover, it is more representative of brain tumor genomic alterations than plasma, and it can identify gene mutations such as *EGFR* [12].

In this study, we used a highly sensitive and specific method to analyze *EGFR* mutations in ctDNA with NGS technology. Here, we present a case of a MC patient, who was provided treatment based on analysis of *EGFR* mutations in ctDNA of their CSF.

## Case presentation

According to the kit instructions, cell free DNA (cfDNA) and genomic DNA (gDNA) were extracted by the QIAamp Circulating Nucleic Acid kit (QIAGEN) Circulating Nucleic Acid Kit extraction kit and blood genome DNA extraction kit, respectively. The CSF samples were taken from a − 80 °C refrigerator and thawed at room temperature before extraction (8 ml CSF and 200 μl cell suspension). It was necessary to control the pressure of the vacuum pump (the calibration of the vacuum pump was first set to maximum and then slowly set to 0) to ensure that the CSF samples were integrated with the adsorption column. Before the sample library was constructed, the concentration and size of cfDNA fragments were quantitatively detected by Qubit 3 and 2100-H. The KAPA Hyper Prep Kit was used to construct the precapture (terminal repair, A-ESRiT connector (15 μM), connection and PCR amplification). The capture of the target area was based on chip hybridization and liquid hybridization capture products from the SureSelect QXT rapid hybridization kit (Agilent Company). A total of 120 cancer-associated genes were included in the gene panel, which was used for target region capture. The library was then tested by Agilent 2100 and Q-PCR (including size detection of the insertion fragment and concentration calculation of the effective fragment). Lastly, the Illumina NextSeq 500 system was used for sequencing.

After trimming the adapter sequences, and filtering and removing poor quality reads using flexbar software (version 2.7.0, https://sourceforge.net/projects/flexbar/), the clean fastq (the raw data file format of from sequencing) data were generated from the raw data from the NEXT SEQ 500 runs. Then, the clean fastq data was aligned to hg19 (GRCH37) and assembled through BWA-sampe (Burrows Wheeler Aligner software version 0.7.12-r1039, https://sourceforge.net/projects/bio-bwa/files/). The polymerase chain reaction (PCR) duplicates were removed by MarkDuplicates in Picard Tools (version 1.124, http://broadinstitute.github.io/picard/). Variant calling was performed in the CSF (CerebroSpinal Fluid) by GATK (GenomeAnalysisTK version 3.6, https://software.broadinstitute.org/gatk/). All variants were annotated by ANNOVAR (version 20,160,201, http://annovar.openbioinformatics.org/en/latest/). Lastly, variation frequency (> 0.5%) was used to eliminate erroneous base calling and generate the final mutations. Manual verification was performed by IGV (Integrative Genomics Viewer version 2.3.72, http://software.broadinstitute.org/software/igv/).

**Fig. 1:**
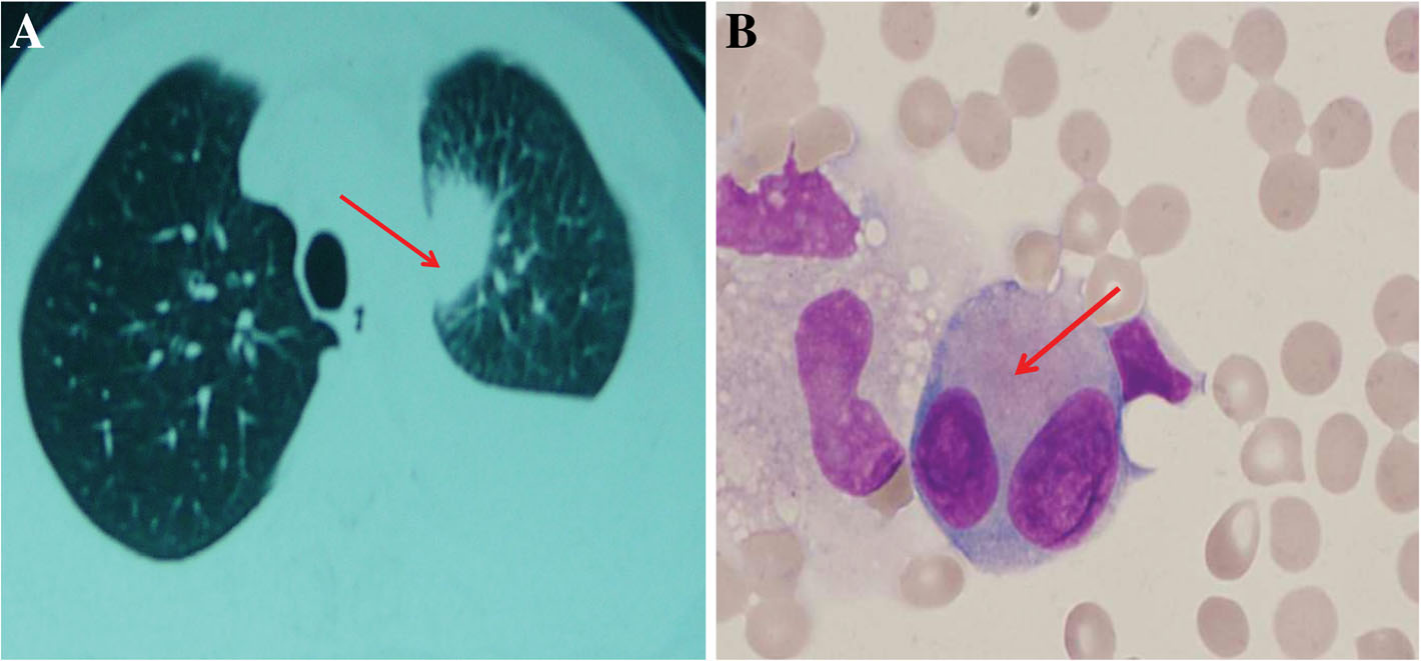
Pulmonary Magnetic Resonance imaging and CSF cytology exams, (Magnification: 1000X) before adjusting the medication based on the NGS. **a** Left lung placeholder before hospital admission. The red arrow identifies the placeholder. **b** Cell types in CSF. CSF showed several tumor cells. Lymphocyte cells accounted for 20% of the cell populations, monocytes accounted for 22%, activated monocytes accounted for 40%, and neutrophils accounted for 18%. The red arrow identifies the tumor cell

**Fig. 2:**
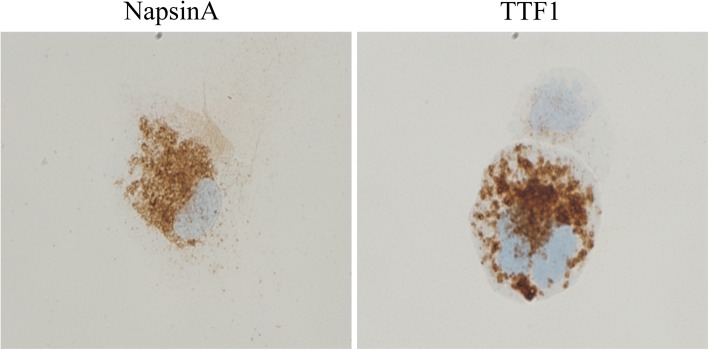
The immunohistochemistry of CSF for NapsinA and TTF1. Magnification: 1000X

**Fig. 3:**
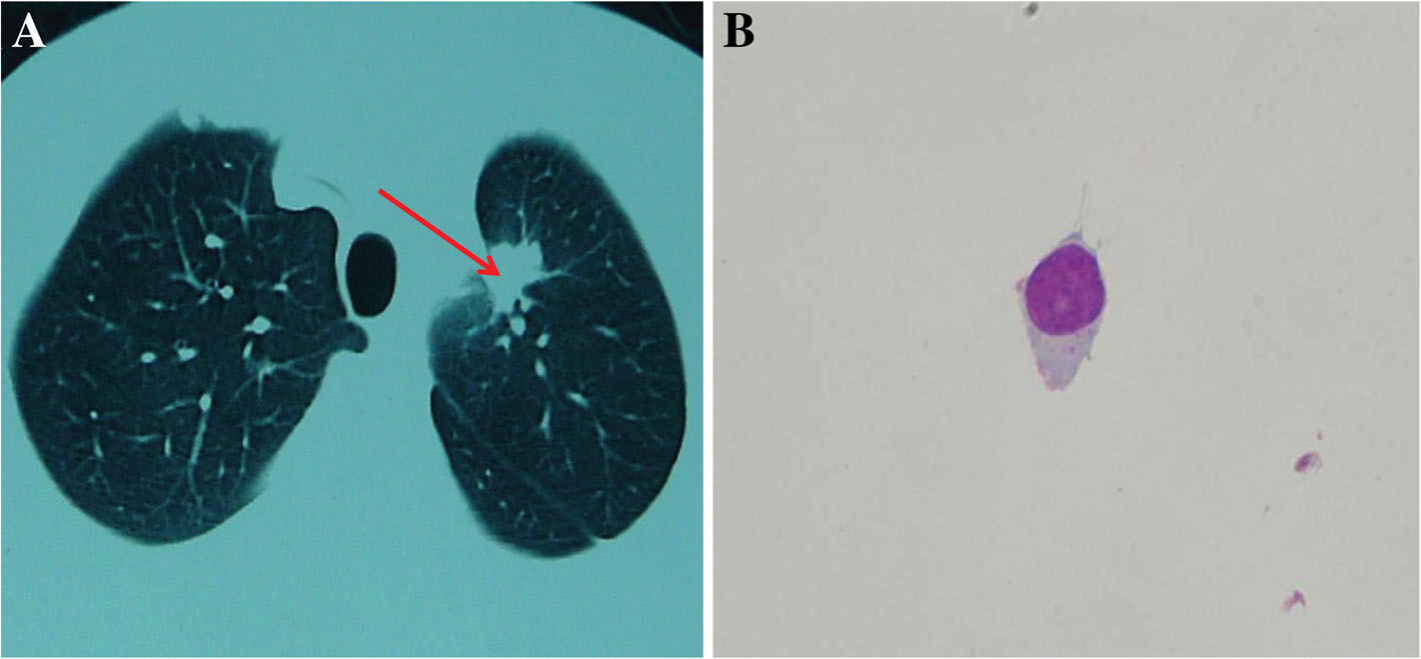
Pulmonary Magnetic Resonance imaging and CSF cytology exams, (Magnification: 1000X) after adjusting the medication based on the NGS. **a** Lesion in the superior lobe of the left lung was significantly reduced after adjusting the targeted drug therapy. The red arrow identifies the placeholder. Figure 2 CSF cytology exams. Magnification: 1000X. **b** Cell types in CSF. CSF showed 17 lymphocytes, 3 monocytes, 2 activated monocytes and 2 neutrophilic granulocytes

Finally, a 19-Del mutation in the exon of the *EGFR* gene was found in gene detection of a patient’s pleural effusion on March 11, 2015. He took 250 mg oral gefitinib, once a day, for 11 months beginning in December 2015. On the 3rd of November 2016, he came to the hospital with dizziness, headache and blurred vision that was transient. He began to take 4 mg oral zoledronic acid, once a month, to prevent bone metastasis. He came to the department of neurology when his neurological symptoms appeared. Pulmonary CT results showed that his left lung appeared as a placeholder (Fig. 1a). A cerebrospinal cytology exam was taken after admission. Several tumor cells could be seen in the cerebrospinal fluid (Fig. 1b). The immunohistochemistry showed positive staining for NapsinA and TTF1 (Fig. 2). The cerebrospinal cytology exam showed that the patient had meningeal carcinomatosis. After that, a lumbar puncture and an intrathecal injection of dexamethasone (5 mg) and methotrexate (10 mg) were conducted twice a week to control meningeal metastases. During this process, the patient was in stable condition and there appeared to be no significant disease progression or side effects of the medicine. At this same time, an appropriate amount of cerebrospinal fluid was preserved for next generation sequencing (NGS). A 19-Del mutation in the exon of the *EGFR* gene (mutation frequency: 30.8%) and a 20-T790 M mutation in the exon of the *EGFR* gene (mutation rate 0.85%) were found in the NGS. In November 2016, the patient discontinued gefitinib and began to take 90–100 mg of oral AZD9291 once a day. After adjusting the medication based on the results of the NGS, the headache of the patient was noticeably reduced, and his condition gradually stabilized. In February 2017, a pulmonary CT showed that the lesion in the superior lobe of the left lung was significantly reduced (Fig. 3a). At this same time, a cerebrospinal cytology exam showed that there were no tumor cells in his cerebrospinal fluid (Fig. 3b). The KPS score increased to 90 from 80 (before treatment), and the ECOG score was still 3.

## Discussion and conclusions

Precision medicine, which relies on genomic information from the tumor, has been applied to cancer treatment [13]. The difficulty of acquiring tumor tissue and the inability to detect ctDNA in plasma leads to a particular challenge for central nervous system tumors [6]. In cancer patients, CSF is the best source of tumor DNA. Presently, some scholars are engaged in related research of tumor DNA in CSF [12, 14]. The development of ctDNA detection has been limited, as there is low detection sensitivity in first generation sequencing (represented by Sanger sequencing). NGS technology has high sensitivity and specificity to distinguish between similar sequences, which makes ctDNA detection possible [15]. However, it is not clear whether a routine lumbar puncture and targeted NGS can be used to identify tumor-associated DNA and provide valuable clinical guidance for treatment response.

It is an important potential application of CSF gene analysis to determine drug resistance mechanisms in patients. Through NGS, we detected ctDNA in the CSF of one MC patient with primary lung cancer, and conducted targeted therapy based on these results. Targeted gene therapy was effective for the primary tumor, however, tumor development of the central nervous system continued to progress. The patient appeared with symptoms in his head after taking oral gefitinib for 11 months. We took CSF from the patient diagnosed with MC to use for NGS. We found a 20-T790 M mutation in the exon of the *EGFR* gene, which is the most common factor seen in resistance to a generation of EGFR tyrosine kinase inhibitors (TKIs) in nonsmall cell lung cancer [16]. The mutation rate was 0.85%, which suggests that the patient had resistance to gefitinib. Recently, there have been several EGFR TKIs approved, such as, first-generation gefitinib and erlotinib, second-generation afatinib, and third generation osimertinib. AZD9291 is an irreversible third generation EGFR TKI, which showed a high response rate in patients whose tumors harbored *EGFR*-T790 M and the capability to penetrate the blood-brain barrier [17, 18]. The condition of the patient improved after oral administration of AZD9291, which suggests the significance of NGS of CSF ctDNA in disease assessment and medication guidance. However, it is worth mentioning that a 19 deletion mutation in the exon of the *EGFR* gene is still present, which suggests that targeted medicines possess a certain sensitivity. After taking oral AZD9291, the patient’s clinical symptoms improved.

In conclusion**,** our study confirmed that ctDNA can be detected in the CSF from MC patients. In addition, we used NGS technology and found a 20-T790 M in the exon of the *EGFR* gene in ctDNA from CSF, which may be helpful in providing drug treatment guidance.

## Abbreviations

cfDNA: cell free DNA
CSF: Cerebrospinal fluid
ctDNA: circulating tumor DNA
EGFR: Epidermal growth factor receptor
gDNA: genomic DNA
MC: Meningeal carcinomatosis
NGS: Next generation sequencing
PCR: Polymerase chain reaction
TKIs: Tyrosine kinase inhibitors
